# Chemical Compositions and Antioxidant Activities of Polysaccharides from the Sporophores and Cultured Products of *Armillaria mellea*

**DOI:** 10.3390/molecules20045680

**Published:** 2015-03-31

**Authors:** Shanshan Zhang, Xiaoqian Liu, Lihua Yan, Qiwei Zhang, Jingjing Zhu, Na Huang, Zhimin Wang

**Affiliations:** 1 Institute of Chinese Materia Medica, China Academy of Chinese Medical Sciences, Beijing 100700, China; E-Mails: qingshuibaikai@126.com (S.Z.); lianyu1127@126.com (X.L.); zhangqw1955@163.com (Q.Z.); zhujj15@163.com (J.Z.); guofurong52@126.com (N.H.); 2 National Engineering Laboratory for Quality Control Technology of Chinese Herbal Medicines, Beijing 100700, China

**Keywords:** *Armillaria mellea*, polysaccharides, wild sporophores, cultured products, chemical composition, antioxidant activity

## Abstract

*Armillaria mellea* is a traditional Chinese medicinal and edible mushroom. Many cultured products of *A. mellea* have been used to develop commercial medicines in recent years. The chemical composition and activities of the major bioactive chemical components—polysaccharides—may be different because of differences in the raw materials used. Four polysaccharides (SP, CMP, CFBP and CFMP) were obtained from wild sporophores and cultured products (including mycelia, fermentation broth and fermentation mixture) of *A. mellea*. Their yields, carbohydrate contents, monosaccharide compositions, FT-IR spectra, NMR spectroscopy and antioxidant activities were investigated. All of the polysaccharides were composed of xylose, glucose and galactose without protein. Glucose was the dominant monosaccharide in SP, CMP and CFMP, whereas galactose was the dominant monosaccharide in CFBP. SP and CMP showed higher scavenging DPPH^•^ and ABTS^•+^ activities and reducing power among four polysaccharides. The carbohydrate content and corresponding glucose percentage were positive influences on the antioxidant activities, whereas the corresponding xylose and galactose percentage were negative influences. *A. mellea* polysaccharides are potential natural antioxidants. Polysaccharides from cultured products, especially mycelia, are good substitutes for SP and are also potential sources for both dietary supplements and food industries.

## 1. Introduction

Mushrooms have been used as foods and traditional medicines for centuries. In recent decades, more and more mushrooms have been used as crude materials in developing dietary supplements because of their nutritive and non-poisonous properties [1]. Many studies have demonstrated that polysaccharides are a class of active components of mushrooms, and they exhibit various pharmacological activities, including antitumor [2], immunomodulation [3], anticoagulant [4] and antioxidant functions [5,6]. Numerous polysaccharide products from well-known mushrooms have been used as medicines or dietary supplements, such as lentinan from *Lentinus edodes*, krestin from *Trametes versicolor* and GLPS from *Ganoderma lucidum* [7]. Thus, it is necessary to study the polysaccharides from more promising culinary-medicinal mushrooms.

Studies have shown that excess reactive oxygen species (ROS) lead to oxidative stress and cause numerous diseases which seriously threaten human health, such as cardiovascular disease [8] and cancer [9]. To inhibit the over-production of ROS, some synthetic antioxidants are used in the food industries, such as butylated hydroxyanisole (BHA) and butylated hydroxytoluene (BHT) [10]. However, synthetic antioxidants are probably toxic and may cause severe food safety problems after high dosage and long-term treatment [11,12]. Thus, it is essential to find low toxicity or nontoxic natural antioxidants as substitutes for synthetic antioxidants. Polysaccharides are an important source of natural antioxidants.

*Armillaria mellea (Tricholomataceae)*, also known as zhen-mo and honey mushroom in China, is an edible and medicinal mushroom widely distributed in North America, Europe and northeast Asia [13]. The sporophores of *A. mellea* have been used for treating megrim, headache, neurasthenia, insomnia, acroanesthesia, hypertension, epilepsy and nyctalopia in China [14]. However, the practical applications of sporophores are restricted because the wild resources of the sporophores are rare and the cultivation conditions are harsh [15].

*A. mellea* has a strong symbiotic relationship with *Gastrodia elata*, which is a slow growing and expensive traditional Chinese medicine. Studies show that the biological activities and clinical applications of *A. mellea* submerged culture products are similar to those of *G. elata* [16]. Thus, many cultured products have been used to develop commercial medicines in recent years. For example, fermentation broth, the mycelia-free filtrates of submerged culture products, was used in Naoxinshu oral liquids. Mycelia (separated from the fermentation end products by filtration) and fermentation mixture (the fermentation end products consisting of mycelia and fermentation broth) were used in Mihunjun tablets, Yuntongding capsules and Yinmi tablets [14].

Polysaccharides are major chemical components of *A. mellea* with numerous bioactivities [17,18,19,20,21]. Submerged culture is an efficient method for production of polysaccharides from many mushrooms [22]. However, to the best of our knowledge, there is no information published about the comparison of polysaccharides from wild sporophores and submerged cultured products (mycelia, fermentation mixture and fermentation broth) with respect to the chemical compositions and activities.

In this paper, to demonstrate the differences and similarities of polysaccharides from wild sporophores and cultured products of *A. mellea* with regards to the chemical compositions and biological activities, polysaccharides were firstly extracted from wild sporophores, mycelia, fermentation mixture and fermentation broth. Then, the chemical compositions of these polysaccharides, including the carbohydrate contents, protein contents and monosaccharide compositions were determined using ultraviolet spectroscopy (UV) and gas chromatography (GC) analysis, and the anomeric configuration and sugar residues of four polysaccharides were investigated using Fourier-transform infrared (FT-IR) and nuclear magnetic resonance (NMR) analysis. Furthermore, the antioxidant activities of four polysaccharides were evaluated by three models *in vitro*. In addition, the relationships between the chemical compositions and the antioxidant activities of the polysaccharides were discussed.

## 2. Results and Discussion

### 2.1. Chemical Composition of SP, CMP, CFMP and CFBP

Four polysaccharides (named SP, CMP, CFMP and CFBP) were extracted from the *A. mellea* sporophores, mycelia, fermentation mixture and fermentation broth by boiling-water extraction, centrifugation, ethanol precipitation and deproteinization, respectively.

The yields and carbohydrate contents of these polysaccharides are shown in Table 1. Among the four samples, CFBP showed the highest yield (24.38% ± 3.34%) and lowest carbohydrate content (52.73% ± 3.41%). SP showed a moderate yield (16.89% ± 0.71%) and the highest carbohydrate content (68.48% ± 0.14%). The yield of CFMP was the lowest (13.77% ± 1.03%). The carbohydrate contents of CMP and CFBP were moderate (57.68% ± 0.20%, 60.35% ± 0.16%). There was no significant difference (*p* > 0.05) between CMP and CFMP in the yields and carbohydrate contents. The reason for this result maybe that CFBP is an extracellular polysaccharide, whereas SP and CMP are intracellular polysaccharides. The results suggested that extracellular polysaccharides had higher yield and lower carbohydrate contents than intracellular polysaccharides.

**Table 1:** The polysaccharide yields and carbohydrate contents of SP, CMP, CFMP and CFBP.

Samples	Polysaccharide Yield (%)	Carbohydrate Content (%)
SP	16.89 ± 0.71 ^b^	68.48 ± 0.14 ^a^
CMP	14.20 ± 2.00 ^a^	57.68 ± 0.20 ^b^
CFMP	13.77 ± 1.03 ^a^	60.35 ± 0.16 ^b^
CFBP	24.38 ± 3.34 ^c^	52.73 ± 3.41 ^c^

Values within a column labeled by different superscript letters imply significantly differences (*p* < 0.05); Values are means ± SD (*n* = 3).

The proteins of the four polysaccharides were detected using the biuret and spectrophotometric methods. No significant absorption at 540 nm (biuret method) and at 260 or 280 nm (spectrophotometric method) in the UV-vis spectra of all samples indicated that there was no protein or nucleic acid in the polysaccharides of *A. mellea* [23].

The monosaccharide compositions of the polysaccharides were determined by gas chromatography and compared with monosaccharide standards. The results are shown in Table 2. All of the polysaccharides had the same monosaccharide compositions. The major monosaccharide components were d-xylose, d-glucose and d-galactose, whereas the molar percentages were different for the four polysaccharides. In SP, CMP and CFMP, glucose was the main monosaccharide (55.83%, 46.49% and 44.20%, respectively) and xylose was the least prevalent monosaccharide (9.80%, 19.10% and 20.30%, respectively). The percentages of galactose were similar (34.37%, 34.41% and 35.50%, respectively). In CFBP, glucose was the least prevalent monosaccharide (24.33%), and galactose was the main monosaccharide (43.96%). The percentage of xylose in the polysaccharides of the cultured products was higher than that of the sporophores, whereas the percentage of glucose in the polysaccharides of the cultured products was lower than that of the sporophores. 

**Table 2:** Monosaccharide compositions and molar percentages (%) of SP, CMP, CFMP and CFBP.

Sample	Xylose (%)	Glucose (%)	Galactose (%)
SP	9.80	55.83	34.37
CMP	19.10	46.49	34.41
CFMP	20.30	44.20	35.50
CFBP	31.71	24.33	43.96

**Figure 1:**
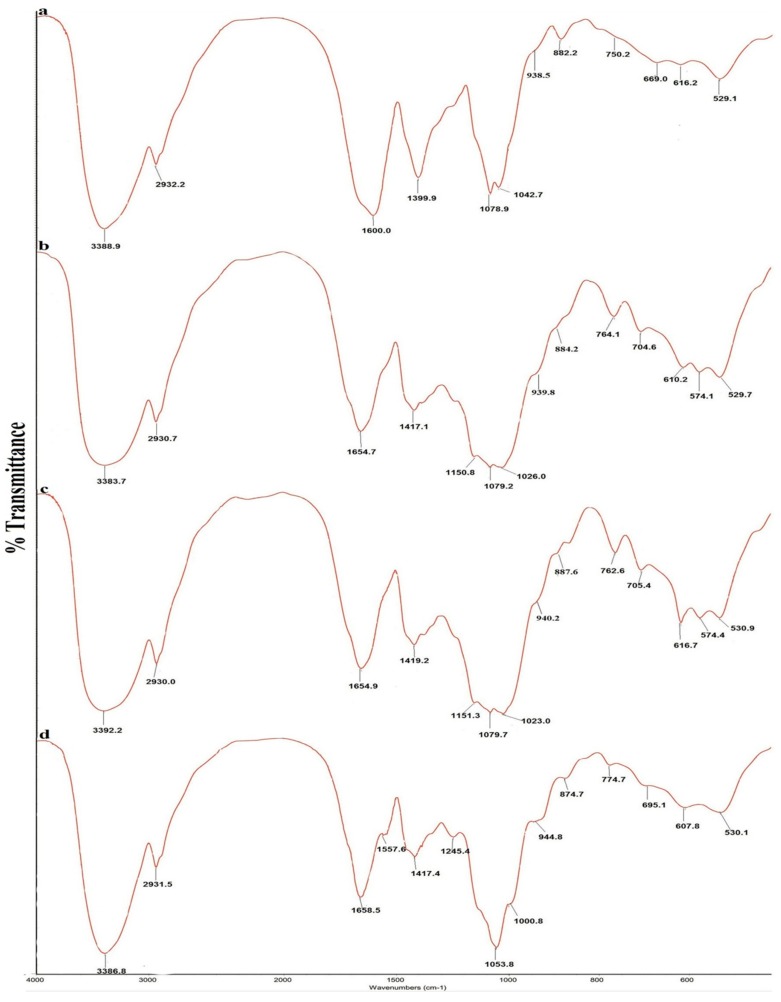
FT-IR spectra of four polysaccharides from *Armillaria mellea*. (a) SP; (b) CMP; (c) CFMP; (d) CFBP.

The FT-IR spectra of the four polysaccharides are shown in Figure 1, and the absorption bands of the four polysaccharides are shown in Table 3. All spectra were similar and showed characteristic polysaccharide bands. The strong and wide absorption of approximately 3390 cm^−1^ was attributed to O-H stretching vibrations. The weak absorption band of approximately 2930 cm^−1^ was attributed to C-H stretching vibrations of methylene [24,25]. The bands of approximately 1600–1654 cm^−1^ were due to bound water [26]. A sharp absorption band of approximately 1410 cm^−1^ was attributed to methylene C-H bending vibrations [24]. A strong and wide absorption band from 1000 cm^−1^ to 1150 cm^−1^ was attributed to the characteristic stretching vibration of C-O-C and the C-O-H bending vibration [26]. The characteristic band of approximately 940 cm^−1^ was attributed to D-glucopyranose ring antisymmetrical vibration [24,27]. The peak at around 880 cm^−1^ was attributed to the D-galactopyranose or β-D-glucopyranose bending vibration [26,27]. The characteristic band atapproximately 770 cm^−1^ was attributed to D-glucopyranose ring symmetrical ring vibration [24]. The FT-IR spectra indicate that the four *A. mellea* polysaccharides contain more β-configuration monosaccharides.

**Table 3:** FT-IR absorption bands of SP, CMP, CFMP and CFBP.

Structural Characteristics	Absorption (cm^−1^) ^a^			
	SP	CMP	CFMP	CFBP
O-H stretching vibration	3388.9	3383.7	3392.2	3386.8
C-H stretching vibration	2932.2	2930.7	2930.0	2931.5
bound water	1600.0	1654.7	1654.9	1658.5
C-H bending vibration	1399.9	1417.1	1419.2	1417.4
C-O-C stretching vibration	1078.9	1140.8, 1079.2	1151.3, 1079.7	1053.8
C-O-H bending vibration	1042.7	1026.0	1023.0	1000.8
antisymmetrical ring vibration	938.5	939.8	940.2	944.8
d-galactopyranose/β-d-glucopyranose bending vibration	882.2	884.2	887.8	874.7
symmetrical ring vibration	750.2	764.1	762.6	774.7

^a^: The FT-IR spectra of four polysaccharides were determined using a Fourier transform infrared spectrophotometer over the frequency range of 400–4000 cm^−1^.

**Figure 2:**
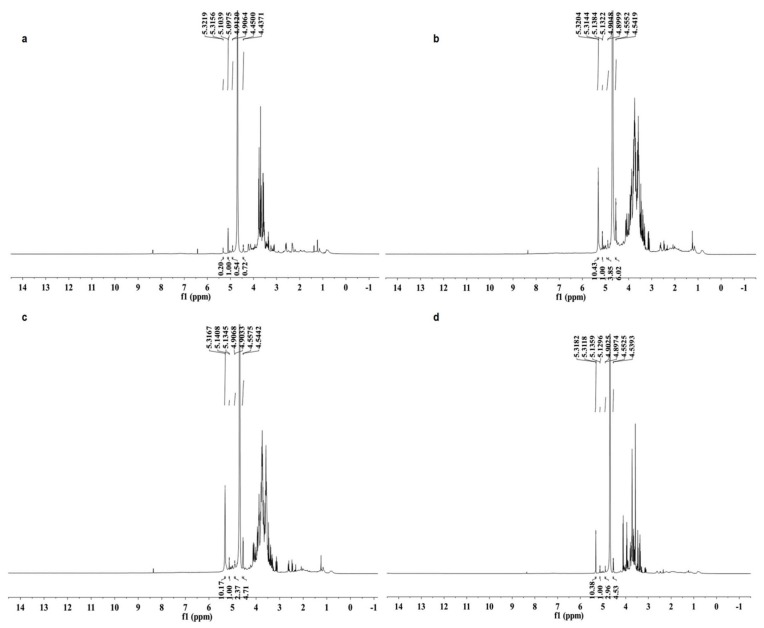
^1^H-NMR spectra (600 MHz, D_2_O, 27 °C) of four polysaccharides from *Armillaria mellea*. (**a**) SP; (**b**) CMP; (**c**) CFMP; (**d**) CFBP.

The ^1^H-NMR spectra of the four polysaccharides are shown in Figure 2. The ^1^H signals at δ 4.90–5.32 ppm with coupling constants (^3^*J*_1,2_) less than 4.0 Hz and ^1^H signal at δ 4.44–4.45 ppm with ^3^*J*_1,2_ larger than 7.0 Hz indicated that the glycosidic linkages of monosaccharides are both α and β configurations in the four polysaccharides [28]. The chemical shifts from 3.29 to 4.23 ppm were assigned to protons of C2 to C5 (C6) of the glycosidic ring [29].

**Figure 3:**
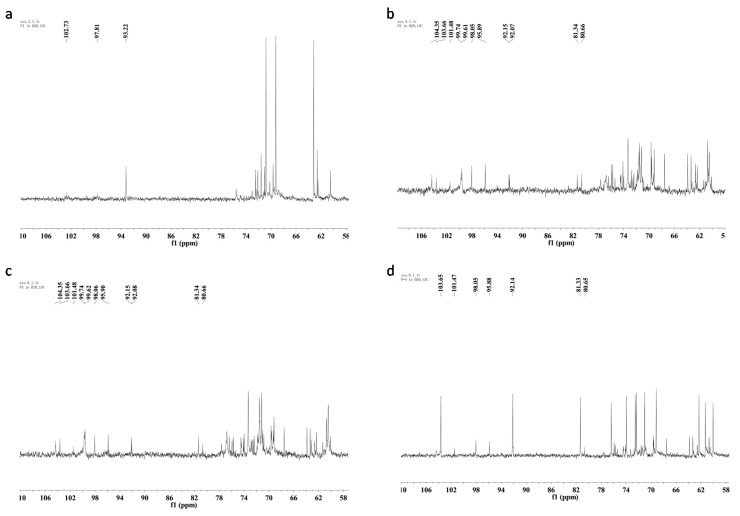
^13^C-NMR spectra (150 MHz, D_2_O, 27 °C) of four polysaccharides from *Armillaria mellea*. (**a**) SP; (**b**) CMP; (**c**) CFMP; (**d**) CFBP.

The ^13^C-NMR spectra of the four polysaccharides are illustrated in Figure 3. SP showed a different ^13^C-NMR spectrum from other samples. Only one obvious signal appeared in the anomeric carbon resonances region at δ 93.2. The major anomeric carbons signals of the other three polysaccharides at δ 92.1–103.7 ppm suggested the presence of both α and β anomeric configurations [29].

^1^H-NMR and ^13^C-NMR spectra of CMP and CFMP are similar, which conforms to the monosaccharide composition and FT-IR analysis. SP and CFBP show different NMR spectra than CMP and CFMP, which may be caused by the different linkage types, the different degree of branching, or the different purity of the crude polysaccharides. All four polysaccharides have both α- and β-configuration sugar residues and the β-configuration is more prevalent.

### 2.2. DPPH Radical Scavenging Activity

**Figure 4:**
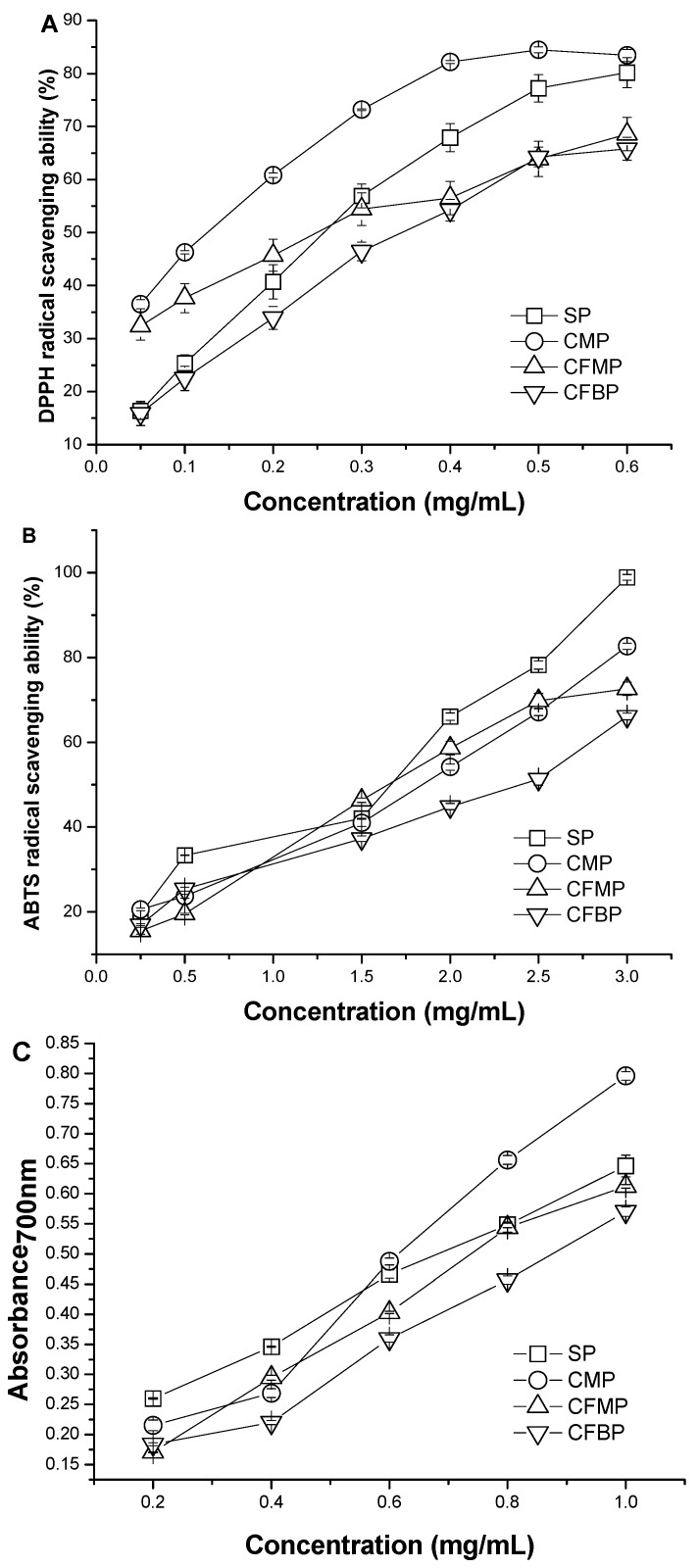
Antioxidant activities of SP, CMP, CFMP and CFBP by scavenging ability for (**A**) DPPH radicals; (**B**) ABTS radicals; and (**C**) reducing power.

The scavenging abilities of *A. mellea* polysaccharides for DPPH radical are shown in Figure 4A, and the IC_50_ values of the samples are listed in Table 4. In general, the antioxidant activity is expressed as the IC_50_ values of the samples. The lower the IC_50_ value is, the higher the antioxidant activity is. The IC_50_ values of the four polysaccharides were in the order of CFBP > SP > CFMP > CMP. The IC_50_ value of SP (223.7 ± 0.011 μg/mL) was similar to the IC_50_ value of CFMP (206.0 ± 0.036 μg/mL) (*p* > 0.05). Although the scavenging activities of the four polysaccharides were lower than that of the positive controls (7.032 ± 0.006 μg/mL of BHA, 7.296 ± 0.004 μg/mL of VC), the four polysaccharides also demonstrated good scavenging activity for DPPH radicals. All four polysaccharides had scavenging activities in a concentration-dependent manner at a concentration range from 0.05 mg/mL to 0.60 mg/mL. At the low concentration of 0.05 mg/mL, the scavenging activities of the four polysaccharides were in the order of CMP > CFMP > SP > CFBP. At the high concentration of 0.60 mg/mL, the scavenging activities were in the order of CMP > SP > CFMP > CFBP, and the scavenging rate of CMP and SP reached more than 80%. The results indicated that polysaccharides from *A. mellea* had an effect on scavenging DPPH radicals, and CMP showed better scavenging activity for DPPH radicals than the other polysaccharides.

**Table 4:** The IC_50_ values of BHA, VC and the four *A. mellea* polysaccharides for DPPH radicals, ABTS radicals and reducing power.

Sample	IC_50_ Values (μg/mL)		
	DPPH^•^	ABTS^•+^	FRAP
VC	7.032 ± 0.006	29.82 ± 0.021	13.13 ± 0.070
BHA	7.296 ± 0.004	15.79 ± 0.107	5.867 ± 0.075
SP	223.7 ± 0.011 ^b^	885.7 ± 0.015 ^a^	693.7 ± 0.009 ^b^
CMP	103.7 ± 0.003 ^a^	1348 ± 0.040 ^b^	552.0 ± 0.011 ^a^
CFMP	206.0 ± 0.036 ^b^	1420 ± 0.058 ^b^	768.2 ± 0.004 ^c^
CFBP	322.3 ± 0.030 ^c^	2118 ± 0.096 ^c^	996.3 ± 0.009 ^d^

Values within a column labeled by different superscript letters imply significantly differences (*p* < 0.05); Values are means ± SD (*n* = 3). The sequence of the letters in the alphabet means the order of the antioxidant ability of four samples.

### 2.3. ABTS Radical Scavenging Activity

The scavenging activities of *A. mellea* polysaccharides on ABTS^•+^ are shown in Figure 4B, and the IC_50_ values of the samples are listed in Table 4. The scavenging activities of the four polysaccharides were lower than that of the positive controls (29.82 ± 0.021 μg/mL of BHA, 15.79 ± 0.107 μg/mL of VC). The IC_50_ values of the four polysaccharides were in the order of CFBP > CFMP > CMP > SP. The IC_50_ value of CMP (1348 ± 0.040 μg/mL) was similar to the value of CFMP (1420 ± 0.058 μg/mL) (*p* > 0.05). The ABTS radical scavenging activity of all samples was also concentration dependent. At the high concentration of 3 mg/mL, the scavenging activities of the four polysaccharides were in the order of SP > CMP > CFMP > CFBP, and the scavenging rate of SP was almost 100%. The results indicated that *A. mellea* polysaccharides had an effect on scavenging ABTS radicals at high concentrations, and SP showed better scavenging activity for ABTS radicals than the other polysaccharides.

### 2.4. Ferric-Reduction Antioxidant Power

The reducing powers of *A. mellea* polysaccharides are shown in Figure 4C, and the IC_50_ values of the samples are listed in Table 4. The ferric-reducing antioxidant powers of the samples were directly proportional to their concentrations within the test dosage range. At a concentration of 0.20 mg/mL, the reducing powers of the four polysaccharides were in the order of SP > CMP > CFBP > CFMP, and at a concentration of 3 mg/mL, the reducing powers of the four polysaccharides were in the order of CMP > SP > CFMP > CFBP. The IC_50_ values of the four polysaccharides were in the order of CFBP > CFMP > SP > CMP. The results revealed that the four polysaccharides from *A. mellea* had an effective reducing power, and CMP showed better reducing power than other polysaccharides.

### 2.5. Discussion of the Chemical Composition-Activity Relationship

The antioxidant activity of polysaccharides can be affected by their chemical composition, contents, conformation and molecular size [30], whereas the chemical compositions and contents depend on the cultivation method, environmental conditions [31] or different species [32] of raw materials. In our study, the difference in antioxidant activities of the four polysaccharides may be affected by the different chemical compositions of the different species of raw materials (sporophores and cultured products).

**Figure 5:**
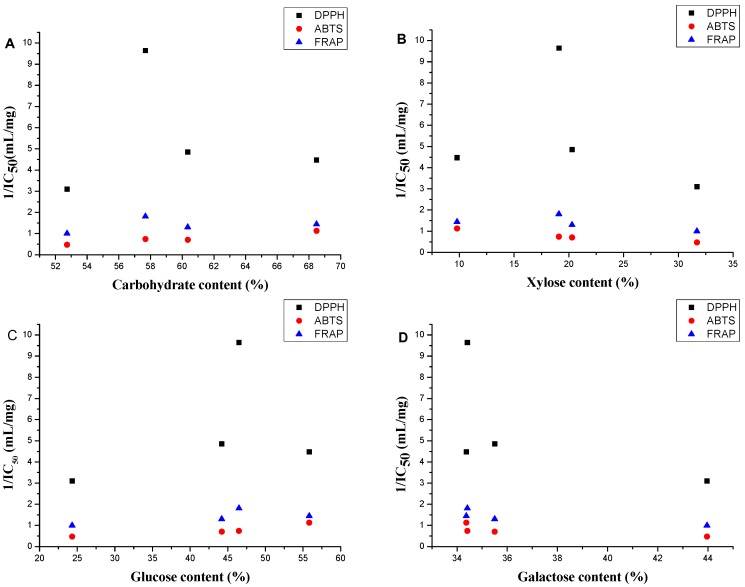
Scatter diagram for the antioxidant 1/IC_50_ values and (**A**) carbohydrate content, (**B**) xylose percentage; (**C**) glucose percentage and (**D**) galactose percentage in four *A. mellea* polysaccharides.

The scatter diagrams for the antioxidant activity 1/IC_50_ values and the chemical compositions of the four polysaccharides are shown in Figure 5 to show the relation between the antioxidant activity and the chemical composition. The polysaccharides with carbohydrate content over 58% had better antioxidant activities in the three models (Figure 5A). This phenomenon can be explained by the fact that polysaccharides with higher numbers of hydroxyls have better antioxidant activity [33]. Polysaccharides with higher corresponding molar percentage of glucose and lower corresponding molar percentage of xylose and galactose had better antioxidant abilities in the three models (Figure 5B–D).

Correlation analysis was used to further analyzing the relationships between the chemical compositions and the antioxidant activities (IC_50_ values) of the four polysaccharides. The correlation coefficients have been shown in Table 5 and the linear regression equations have been shown in Table 6.

**Table 5:** Pearson correlation coefficients between carbohydrate content/monosaccharide percentage and antioxidant activities (IC_50_).

	DPPH	ABTS	FRAP
DPPH	1.000	0.580 *	0.931 **
ABTS	0.580 *	1.000	0.799 **
FRAP	0.931 **	0.799 **	1.000
Carbohydrate content	−0.671 *^a^	−0.915 **	−0.667 *^a^
Xylose percentage	0.682 *	0.796 **	0.797 **
Glucose percentage	−0.616 *	−0.993 **	−0.803 **
Galactose percentage	0.830 **	0.844 **	0.883 **

** represents statistical significance at *p* < 0.01; * represents statistical significance at *p* < 0.05; ^a^ Calculated without SP.

**Table 6:** The linear regression equations of the relation between carbohydrate content/monosaccharide percentage (X) and antioxidant activities (Y).

		DPPH	ABTS	FRAP
	
Carbohydrate content		Y = −1.076ln(X) + 4.560 ^a^	Y = −3.922ln(X) + 17.50	Y = −2.156ln(X) + 9.480 ^a^
Xylose percentage		Y = 0.006X + 0.090	Y = 0.039X + 0.696	Y = 0.015X + 0.461
Glucose percentage		Y = −0.04X + 0.396	Y = −0.036X + 3.008	Y = −0.011X + 1.230
Galactose percentage		Y = 0.017X − 0.426	Y = 0.093X − 1.967	Y = 0.037X − 0.616

^a^: Calculated without SP.

Significant correlations between DPPH, ABTS and FRAP were observed (*p* < 0.05, *p* < 0.01) indicating that these three assays showed consistent results for the polysaccharides of four *A. mellea* samples [34]. Carbohydrate content showed significant correlation with antioxidant activities in the ABTS radical assay (*p* < 0.01), whereas it did not show good correlation with antioxidant activities in the DPPH radical assay (r = −0.257) and FRAP assay (r = −0.499). With the exception of SP, a strong correlation was found between carbohydrate content and the antioxidant activities (*p* < 0.05) in DPPH radical assay and FRAP assay. The xylose percentages, the glucose percentages and the galactose percentages of four polysaccharides also showed strong correlation with antioxidant activities in three antioxidant activity evaluation methods (*p* < 0.05). These results indicated that the carbohydrate content and the corresponding glucose percentage had a positive influence on the activity, whereas the influence of the corresponding xylose and galactose percentages were negative.

## 3. Experimental Section

### 3.1. Materials and Reagents

The wild sporophores of *Armillaria mellea* were collected in a forest (Hulin, Heilongjiang Province, China) in August 2013. A voucher specimen has been deposited at the Institute of Chinese Materia Medica, China Academy of Chinese Medical Sciences. Samples were ground and passed through 40 mesh screen after drying. The cultured products (mycelia, fermentation mixture and fermentation broth) were cultured on liquid medium obtained from Kang Xin Pharmaceuticals Co., Ltd. (Wenshui, Shanxi Province, China). Mycelia and fermentation broth were obtained from the final liquid culture medium by filtration, respectively. The precipitation (A) was dried and grounded to give the mycelia. The supernatant liquid was concentrated to paste (B) by evaporation to achieve the fermentation broth. A and B were combined and dried to powders to obtained the fermentation mixture. The fermentation mixture was the main raw materials of most of *A. mellea* commercial medicines. Monosaccharide standards were purchased from the National Institute for the Control of Pharmaceutical and Biological Products (Beijing, China). Trifluoroacetic acid (TFA), trichloroacetic acid, pyridine and ascorbic acid (VC) were purchased from Sinopharm Chemical Reagent Co., Ltd. (Shanghai, China). Butylated hydroxyanisole (BHA) and bovine serum albumin (BSA) were purchased from Aladdin Reagent Co., Ltd. (Shanghai, China). 1,1-Diphenyl-2-picrylhydrazyl (DPPH) was purchased from Sigma Chemical Co. (St. Louis, MO, USA). Trimethylchlorosilane (TMCS), 1,1,1,3,3,3,-hexamethyl-disilazane (HMDS), L-cysteine methyl ester hydrochloride and 2, 2'-azinobis-(3-ethylbenzothiazoline-6-sulfonic acid ammonium salt) (ABTS) were purchased from Tokyo Chemical Industrial Co., Ltd. (Shanghai, China). All other chemical regents were of analytical grade.

### 3.2. Preparation of Polysaccharides

The extraction method of polysaccharides was the same for the sporophores, mycelia and fermentation mixture, and it was performed as follows: the powdered sample was extracted with distilled water (1:50, W/V) at 100 °C three times for 2 h each time. The residue was removed by filtration. The filtrates were combined and then concentrated in a rotary evaporator under reduced pressure at 45 °C. After the condensate was cooled, 95% ethanol was added to the solution slowly until a final concentration of 50% (V/V) and was kept overnight at 4 °C. The precipitate was separated by centrifugation (12,000× *g* for 10 min), and 95% ethanol was added to the supernatant to reach a final concentration of 70% (V/V) and was then kept overnight at 4 °C. Then, the solution was centrifuged (12,000× *g* for 10 min) to obtain the precipitate. The combined precipitate was washed twice with anhydrous ethanol and then dissolved in distilled water. The proteins were removed by the Sevag method [35]. Dry polysaccharides were then obtained by lyophilization. The polysaccharides from the sporophores, mycelia and fermentation mixture were named SP, CMP and CFMP, respectively.

After the fermentation broth was dissolved in an adequate amount of water, the 95% ethanol was added to the solution slowly until a final concentration of 50% (V/V). The sequent process was the same as that of other polysaccharides. Then, the polysaccharide from the fermentation broth was obtained and it was named CFBP. The polysaccharide yield (*Y*) was calculated by Equation (1):

(1) $$Y\text{ (\%, w/w) = }\frac{\text{weight of polysaccharides (g)}}{\text{weight of raw material (g)}}\text{ × 100}$$

### 3.3. Monosaccharide Composition Analysis

The carbohydrate content was determined by the phenol-sulphuric acid method with glucose as the standard [36]. The protein was detected by the biuret method (540 nm) with bovine serum albumin as the standard [37] and by a spectrophotometric method (260 nm and 280 nm). The monosaccharide compositions of the polysaccharides were measured according to the reported method with some modification [38]. A two milligram polysaccharide sample was hydrolysed in 2 mL of 2 M trifluoroacetic acid (TFA) at 110 °C for 2 h. The TFA was then removed using a rotary vacuum evaporator at 45 °C. The residue was reacted with anhydrous pyridine (100 μL) and 0.06 M L-cysteine methyl ester hydrochloride-pyridine (100 μL) at 60 °C for 1 h. Then, 75 μL 1,1,1,3,3,3-hexamethyl-disilazane (HMDS) and 75 μL trimethylchlorosilane (TMCS) were added, and the resultant reaction mixture was kept at 60 °C for another 30 min. The precipitate was separated by centrifugation (12,000× *g* for 10 min), and the supernatant was obtained. The trimethylsilyl-l-cysteine derivatives were analysed by gas chromatography (GC) on an Agilent 7890A instrument (Agilent Technologies, Palo Alto, CA, USA) equipped with an HP-5 capillary column (0.25 μm × 0.25 mm × 30 m) and a flame ionization detector.

The operation was performed using the following conditions. The column temperature was maintained at 230 °C for 30 min. The flow rate of the N_2_ carrier gas was 1.0 mL/min. The injector temperature was 250 °C. The detector temperature was 250 °C, and the split ratio was set to 20:1. Monosaccharide standards (d-glucose, d-xylose, d-galactose, d-rhamnose, l-rhamnose, d-fructose, l-fructose, d-mannose, d-ribose, d-arabinose and d-glucuronic acid) were prepared according to the above derivatization method and subjected to GC analysis.

### 3.4. Fourier-Transform Infrared (FT-IR) Spectra Analysis

The FT-IR spectra of the four polysaccharides were obtained using a Nicolet 5700 FT-IR spectrometer (Thermo Co., Waltham, MA, USA). The polysaccharides were mixed with KBr powder and pressed into pellets for FT-IR measurement within the frequency range of 4000 to 400 cm^−1^.

### 3.5. Nuclear Magnetic Resonance (NMR) Analysis

Thirty milligrams of sample was dissolved in D_2_O (0.55 mL, 99.9%), freeze-dried, and then redissolved in D_2_O (0.55 mL, 99.9%). The ^1^H-NMR and ^13^C-NMR spectra were measured in an NMR 5 mm tube using an Avance 600 spectrometer (Bruker, Billerica, MA, USA) ^1^H chemical shifts were referenced to the HDO resonance at δ 4.69 ppm (27 °C) as internal standard. ^13^C chemical shifts were determined in relation to TMS (tetramethylsilane, δ 0.00 ppm) as external calibration.

### 3.6. Antioxidant Activity Assays in Vitro

#### 3.6.1. DPPH Radical Scavenging Assay

The DPPH radical scavenging activity of the polysaccharides was measured according to the reported method [39] with a slight modification. Several 2 mL polysaccharide samples of different concentrations (0.05–0.60 mg/mL) were added to 2 mL DPPH solution (0.14 mM in ethanol). Then, the mixture was incubated for 30 min at room temperature in the dark. The absorbance was measured at 517 nm. VC and BHA were used as positive controls. The scavenging rate was calculated by Equation (2). The value of IC_50_ (the extract concentration providing 50% of radicals scavenging activity) was calculated by probit analyses:

(2) $$\text{Scavenging rate (\%) =}\left[1-\frac{A_{1}-A_{2}}{A_{0}}\right]\text{ × 100}$$

where A_0_ is the absorbance of the radical solution without sample, A_1_ is the absorbance of the sample mixed with radical solution, and A_2_ is the absorbance of the sample without radical solution.

#### 3.6.2. ABTS Radical Scavenging Assay

The ABTS radical scavenging activity of the polysaccharides was measured according to the reported method [40] with a slight modification. The ABTS radical cation (ABTS^•+^) was generated by mixing 5 mL of 7 mM ABTS solution with 0.88 mL of 140 mM potassium persulphate and then diluting the potassium persulphate to a final concentration of 2.45 mM with methanol and leaving the mixtures to incubate in the dark at room temperature for 12–16 h. The ABTS^•+^ solution was diluted to an absorbance of 0.7 ± 0.02 at 734 nm prior to use. The ABTS^•+^ solution (2.85 mL) was then added to the sample solutions (0.15 mL) with various concentrations (0.25–3.00 mg/mL). The mixture was incubated for 10 min at room temperature in the dark. The absorbance was measured at 734 nm. VC and BHA were used as positive controls. The radical scavenging rate was calculated by Equation (2). The value of IC_50_ (the extract concentration providing 50% of radical scavenging activity) was calculated by probit analyses.

#### 3.6.3. Ferric-Reducing Antioxidant Power Assay (FRAP)

The reducing power of the polysaccharides was measured by the method of Oyaizu [41] with some modifications. Several 1 mL polysaccharide samples with different concentrations (0.20–1.00 mg/mL) were mixed with 0.5 mL of 0.2 M phosphate buffer (pH 6.6) and 1.5 mL potassium ferricyanide (1%, w/v). The mixture was incubated at 50 °C for 20 min in the dark. Then, 1 mL trichloroacetic acid (10%, w/v) was added to the mixture to terminate the reaction. Afterwards, the mixture was centrifuged at 4000× *g* for 10 min. Subsequently, 2.5 mL of the supernatant was collected and mixed with 2.0 mL deionized water and 0.5 mL ferric chloride (0.1%, w/v). After incubating at room temperature for 5 min, the absorbance was measured at 700 nm. The higher the absorbance of the mixture was, the greater the reducing power of the sample was. VC and BHA were used as positive controls. The value of IC_50_ (the extract concentration providing 0.5 of absorbance) was calculated by the regression equation.

### 3.7. Statistical Analysis

All assays were performed in triplicate, and the data were recorded as the means ± standard deviations (SD). SPSS version 16.0 software was used for the statistical analysis (SPSS Inc., Chicago, IL, USA). One-way analysis of variance (ANOVA) was used to determine the differences among the sample results. Significant differences were analysed using the Student-Newman-Keuls test. A *p* value of less than 0.05 was considered to be significant. The Pearson correlation coefficient was used to evaluate the correlations between the chemical composition and the activity. Linear regression analysis was used calculate the linear regression equation.

## 4. Conclusions

The objective of our study was to evaluate and compare the chemical compositions and antioxidant activities of four different sources of *A. mellea*. Hence, four polysaccharides, which named SP, CMP, CFMP and CFBP, were extracted from the wild sporophores, cultured mycelia, fermentation mixture and fermentation broth of *A. mellea*, respectively. All of the polysaccharides were heteropolysaccharides that were composed of D-xylose, D-glucose and D-galactose in different molar ratios without protein. Glucose was the dominant monosaccharide in SP (55.83%), CMP (46.49%) and CFMP (44.20%), and galactose was the dominant monosaccharide in CFBP (43.96%). The contents of xylose in the polysaccharides of cultured products (19.10% in CMP, 20.30% in CFMP and 31.71% in CFBP) were much higher than that of SP (9.8%). SP and CFBP have different NMR spectra with CMP and CFMP, which may be caused by the different linkage types, the different branch degree or the different purity of the crude polysaccharides.

CMP had excellent antioxidant activity (IC_50_ values of 103.7 μg/mL in DPPH, 1,348 μg/mL in ABTS and 552.0 μg/mL in FRAP) that could match SP (IC_50_ values of 223.7 μg/mL in DPPH, 885.7 μg/mL in ABTS and 693.7 μg/mL in FRAP). CFMP showed moderate antioxidant activity (IC_50_ values of 206.0 μg/mL in DPPH, 1420 μg/mL in ABTS and 768.2 μg/mL in FRAP). The antioxidant activity of CFBP was the lowest among the four polysaccharides, but it showed good antioxidant activity at high concentrations. Above all, *A. mellea* polysaccharides can be used as potential natural antioxidants. Polysaccharides from cultured products, especially mycelia, are good substitutes for SP and also potential sources for both dietary supplements and food industries.
